# AAA WORKFORCE CHALLENGES AND THEIR IMPACT ON OLDER ADULTS: RESULTS FROM A NATIONAL POLL AND AGENCY INTERVIEWS

**DOI:** 10.1093/geroni/igac059.296

**Published:** 2022-12-20

**Authors:** Elizabeth Blair, Traci Wilson

**Affiliations:** USAging, Washington, District of Columbia, United States; USAging, Washington, District of Columbia, United States

## Abstract

Area Agencies on Aging (AAAs) play a critical role in addressing social needs that enable older adults to live independently. In 2022, over 90% of AAAs reported that the number of consumers seeking services and the complexity of consumer needs have increased. At the same time, the nation faces a shortage of direct care workers. Existing AAAs workforce challenges, such as worker shortages and staff burnout, have been exacerbated by the COVID-19 pandemic. We will share the results of a USAging poll of AAAs on the challenges facing their agency and provider workforce, reductions in volunteer staffing, and the impacts of workforce shortages on clients and services provided. Top impacts include clients not receiving the frequency of services needed, if at all. Services most impacted include personal care, respite, and transportation. Presenters will conclude by sharing innovative solutions that AAAs have developed to address these challenges.

